# Effects of plant growth-promoting rhizobacteria on growth indicators and physiological characteristics of *Peucedanum praeruptorum* Dunn leaves

**DOI:** 10.1080/15592324.2023.2203571

**Published:** 2023-04-27

**Authors:** Meng Zhang, Min Hua, Dongqin Guo, Yanbin Xue, Xubing Chen, Lu Rui, Nong Zhou

**Affiliations:** a College of Pharmacy, Dali University, Dali, Yunnan, P.R.China; b College of Food and Biological Engineering/Chongqing Engineering Laboratory for Green Cultivation and Deep Processing of Three Gorges Reservoir Area’s Medicinal Herbs, Chongqing Three Gorges University, Chongqing, P.R.China; c Environmental conservation center, Chongqing Liangjiang Property Management Co. LTD, Chongqing, P.R. China; d School of Design, Chongqing Industry Polytechnic College, Chongqing, P.R.China

**Keywords:** Plant growth-promoting rhizobacteria, *Peucedanum praeruptorum* Dunn, physiological characteristics, growth indicators, growth promotion

## Abstract

As a kind of medicinal plant, *Peucedanum praeruptorum* Dunn has been over-harvested in the wild population, which leads to its artificial cultivation. The present study aims to analyze the effects of different plant growth-promoting rhizobacteria (PGPR) on the growth and physiological characteristics of *P. Praeruptorum* leaves. Compared with the CK, the content of malondialdehyde (MDA) was drastically reduced in the leaves of *P. Praeruptorum* in different treatment groups (*P* < 0.05), and with S6 showing the most significant reduction in MDA content (content was only about 1/3 that of the CK). The indicators of leaf area, length and width were found to be the highest in group S9, reaching a level that is 3.75, 3.08 and 1.48 times higher than those in group CK, respectively. Group S8 has the largest plant height, which is 1.22 times higher than that in group CK. S2 has the largest stem diameter, which is 1.69 times higher than that in group CK. Group S1 has the largest petiole length, which is 1.74 times higher than that in group CK. Group S6 has the largest chlorophyll content, which is 1.63 times higher than that in group CK. Group S2 has the highest content of soluble sugar and soluble protein, which are 2.02 times and 3.82 times higher than those in group CK. Group S9 exhibits the strongest CAT activity, which is 3.71 times higher than that in group CK. S5 exhibits the strongest SOD activity, which is 2.32 times higher than group CK. Group S1 exhibits the strongest POD activity, which is 5.94 times higher than that in group CK. In conclusion, the inoculation with PGPR is effective in improving the growth of *P. Praeruptorum* leaves and their physiological indicators, which provides guidance on the application of PGPR to achieve the high quality and yield of *P. Praeruptorum*.

## 1 Introduction

The roots of *Peucedanum praeruptorum* are referred to as “Peucedani Radix” in Chinese “Qian-hu”.^1^ Containing a variety of different chemical components such as coumarins, flavonoids, and volatile oils, they exhibit various biological activities that contribute to preventing heart failure and myocardial ischemia, reducing blood pressure and alleviating lung inflammation.^2–4^ *P. Praeruptorum*, which was first recorded in Tao Hongjing’s “Famous Doctors’ Records”,^5^ is the primary raw material used to produce a range of Chinese patent medicines, such as Taiji Jizhi Syrup, Cough and Asthma Granules, Xingsu Cough Syrup, Asthmatic Cough and Gas Pills and Tracheitis Pills.^6^ Since the wild resources of *P. Praeruptorum* are in decline at the present time, artificial cultivation is relied on to obtain most of the medicinal materials of *P. Praeruptorum*.^7^ According to a survey, the annual yield of *P. Praeruptorum* amounts to just over 3,000 tons, which is far from enough to meet the annual demand of about 4000 tons.^6^ Also, there are some constraints on cultivation technology, such as early bolting^8^ and substandard quality.^9^ In addition, the abuse of chemical fertilizers and pesticides has resulted in soil pollution and serious nutrient imbalance. Allowing for these issues, it is necessary to achieve the sustainable utilization of *P. Praeruptorum* resources by finding out how to reach a balance between ecological conservation and cultivation quality.

As a class of beneficial bacteria, plant growth-promoting rhizobacteria (PGPR) can be used to promote plant growth and development either directly or indirectly. When attached to plant roots, they exert influence on the growth of plants through their metabolic activities and plant interactions, including decomposition, nutrient cycling conversion and most soil transformation processes.^10–12^ Its growth-promoting mechanism mainly involves the following aspects. Firstly, it promotes the growth of plants by affecting the balance of growth hormones in plants.^13,14^ Secondly, it enhances the disease resistance of plants by suppressing the pathogens.^14^ Lastly, it promotes the transformation and absorption of nutrient elements while improving the soil microenvironment.^13,14^ Due to the growth-promoting characteristics of PGPR, it is applicable as a type of microbial fertilizer to replace the original highly-polluting and pesticides, which not only reduces environmental pollution, but also improves the utilization rate of organic fertilizers.^15,16^ As revealed by Wang Dan et al.^17^, the inoculation with potassium solubilizing bacteria could expand the leaf area, enhance its enzyme activities, and promote the accumulation of osmotic regulatory substances in the leaves of *Paris polyphylla* var. *Yunnanensis*. For this reason, these strains of bacteria are effective in promoting the growth of *Paris polyphylla* var. *Yunnanensis* and in enhancing its performance in stress resistance. According to Jiang Meiyan et al.^18^, the inoculation with *Klebsiella* XI-1 led to a significant increase not only in the indicator of *Angelica dahurica* var. *Formosana* such as plant height, root length and yield, but also in the content of its active ingredients, thereby improving its quality.

At present, the study of *P. Praeruptorum* focuses mainly on the content determination,^19,20^ processing^21,22^ and pharmacological activity.^3,23^ However, there are still few reports on the effects of PGPR on the growth and physiological characteristics of *P. Praeruptorum*. Therefore, the effects of different PGPR on the growth and physiological characteristics of *P. Praeruptorum* were investigated in this paper by conducting pot trials. It was demonstrated that the PGPR was conducive to improving the quality and efficiency of *P. Praeruptorum*. Also, the methods to use them in a safe, scientific and effective way were clarified, which provides a theoretical reference for the cultivation of high-quality *P. Praeruptorum*.

## 2 Materials and methods

### 2.1. Materials

The seeds were derived from the *Peucedanum praeruptorum* Dunn that grew well in Xiangshui Town, Wanzhou District. After being preserved by single plants to ensure homogeneity and stability, they were routinely treated, managed, and identified by Prof. Zhou Nong from Chongqing Three Gorges College as *Peucedanum praeruptorum* Dunn, a member of the Umbelliferae family. In addition, routine management was carried out according to the requirements on outdoor cultivation during the cultivation period. The strains used in the experiments include all the dominant strains of potassium-solubilizing bacteria in the rhizosphere soil that were isolated, cultured and activated in the previous stage of the research^24^ [*Bacillus thuringiensis* (Screened from Changning County, Baoshan, Yunnan, 24°94′20.79″/099°56′52.25″), *Paenibacillus amylolyticu*s (Screened from Yimen County, Yuxi, Yunnan, 24°58′38.89″/102°12′52.17″), *B. polymyxa* (Screened from Changning County, Baoshan, Yunnan, 24°94′20.79″/099°56′52.25″)] organic phosphorus solubilizing bacteria^25^ [*B. mycoides* (Screened from Huidong County, Sichuan Province, 26°23′32.68″/102°57′55.67″), *B. proteolyticu*(Screened from Yulong Naxi Autonomous County, Lijiang, Yunnan, 27°01′94.84″/100°22′01.55″), *B. wiedmanni*i(Screened from Yongping County, Dali, Yunnan 25°21 ’27.85 “/099°23’ 14.09”)] and inorganic phosphorus solubilizing bacteria^25,26^ [*B. aryabhattai*(Screened from Huidong County, Sichuan Province, 26°23′32.68″/102°57′55.67″), *B. aryabhattai*(Screened from Pumiao Town, Baoshan, Yunnan Province 25°02 ’09.58 “/099°04’ 08.31”), *B. cereus*(Screened from Longli County, Guizhou 26°27 ’47.29 “/106°59’ 33.64”)].

To perform the experiment, 9 treatment groups (S1~S9) and a control group were created, with the cultivation substrate sterilized in the absence of inoculation, CK. Specifically, the S1 group was inoculated with *B. thuringiensis*, the S2 group was inoculated with *P. amylolyticus*, the S3 group was inoculated *B. polymyxa*, the S4 group was inoculated with *B. aryabhattai*, the S5 group was inoculated *B. aryabhattai*, the S6 group was inoculated with *B. cereus*, the S7 group was inoculated with *B. mycoides*, the S8 group was inoculated with *B. proteolyticu*, and the S9 group was inoculated with *B. wiedmannii*.

### 2.2. Experimental design

The potted experiment was conducted in March 2021 at the College of Biological and Food Engineering, Chongqing Three Gorges University (Wanzhou District, Chongqing). As a mixture of subsoil, river sand and organic fertilizer (2:1:1), the test soil was obtained from the premise of Chongqing Three Gorges College. After passing through an 8 mm soil sieve, it was subjected to intermittent sterilization at 121°C for 2 h. Then, it was taken out, placed aside for 7 h and sterilized for later use. The plastic pots with a diameter of 15 cm and a height of 18 cm were applied as cultivation containers (repeatedly wiped 3 times with 75% ethanol). The dominant strains were isolated and activated in the early stage of the research, and the inoculum was obtained by adjusting the concentration of the bacterial suspension to 1 × 10^6^CFU/mL with sterile saline. In April 2021, the inoculants were added into the rhizosphere soil of *P. Praeruptorum* seedlings (seed breeding, seedling age 1 year). Then, 30 mL of PGPR bacterial suspension was poured into the holes dug under the drip line of the plants with a length of 8 cm, a width of 5 cm and a depth of 10 cm. Given the same amount of sterile saline, the control group was covered with soil, with the bacteriological agent evenly distributed in the roots of the plants.

### 2.3. Growth indicators measurements

In August, 2021, *P. Praeruptorum* leaves grew very well in each pot, from which those without diseases and pests were randomly selected. Under the condition of not picking leaves, the area, width and length of the leaves growing in the same part of different plants were measured with the assistance of portable laser leaf area meter, while the height, stem diameter and petiole length of the plants were measured by using a steel tape. Each indicator was repeated three times. Finally, the physiological and biochemical indexes of *P. Praeruptorum* leaves as measured against the previous leaf area were used to assess the physiological and biochemical indexes.

### 2.4. Physiological and biochemical indicators measurements

The content of chlorophyll was determined using the method proposed by Shu Zhan et al.^27^ The physiological indicators were finalized using the method developed by Li Hesheng.^28^ The content of soluble protein was determined using the colorimetric method of Coomassie Brilliant Blue G250. The content of malondialdehyde (MDA) and soluble sugar was determined using the thiobarbituric acid heated colorimetric method. The activity of peroxidase (POD) was evaluated using the guaiacol colorimetric method. The activity of catalase (CAT) was evaluated by means of UV spectrophotometry. The activity of superoxide dismutase (SOD) was evaluated using the nitroblue tetrazolium (NBT) method.

### 2.5. Data analysis

The experimental data were processed by using Microsoft Excel 2019, and the statistical analysis was conducted using SPSS25.0 software.

## 3 Results

### 3.1. Effect of inoculation with different PGPR on the growth indicators of P. Praeruptorum

Except for S8 group, the leaf area of *P. Praeruptorum* in all treatment groups was significantly larger than in the CK group (*P* < 0.05), as shown in Table 1. Besides, the plant height, stem diameter, petiole length, leaf length and leaf width were substantially higher in the treatment groups than those in the CK group (*P* < 0.05). The leaf area, length and width were the largest in group S9, which were 3.75 times, 3.08 times and 1.48 times that of the CK group, respectively. The plant height in group S8 was 1.22 times that of group CK. Group S2 had the largest stem diameter, which was 1.69 times that of group CK. Group S1 had the largest petiole length, which was 1.74 times that of group CK. On the whole, the growth indexes of *P. Praeruptorum* were improved to varying degrees by the inoculation of different PGPRs, and the effect of S9 group was more satisfactory.

**Table 1.** Effect of the inoculation with different PGPRs on the growth indicators of P. Praeruptorum.

Treatments	Plant height/cm	Stem thickness/cm	Leaf length/cm	Leaf width/cm	Leaf area/cm2	Petiole length/cm
S1	21.267 ± 0.032cd	0.267 ± 0.0217bc	0.932 ± 0.047bc	5.397 ± 0.206ab	3.510 ± 0.018de	4.067 ± 0.028a
S2	21.167 ± 0.036cd	0.393 ± 0.029a	0.699 ± 0.425cd	3.383 ± 0.175c	2.736 ± 0.036f	2.433 ± 0.166d
S3	23.333 ± 0.025b	0.233 ± 0.247c	0.697 ± 0.129cd	3.700 ± 0.003c	3.700 ± 0.079cd	3.833 ± 0.075ab
S4	21.167 ± 0.036cd	0.333 ± 0.173abc	1.127 ± 0.037b	4.763 ± 0.010abc	3.110 ± 0.093ef	2.333 ± 0.247d
S5	23.100 ± 0.028b	0.267 ± 0.217bc	1.432 ± 0.081a	4.230 ± 0.485abc	4.107 ± 0.014bc	3.167 ± 0.091bc
S6	21.633 ± 0.019cd	0.367 ± 0.157ab	1.600 ± 0.137a	5.293 ± 0.064ab	4.461 ± 0.060b	3.167 ± 0.091bc
S7	21.000 ± 0.044cd	0.267 ± 0.2177bc	0.977 ± 0.122b	4.593 ± 0.030abc	2.858 ± 0.019f	2.167 ± 0.133d
S8	25.033 ± 0.020a	0.267 ± 0.2177bc	0.643 ± 0.085d	4.003 ± 0.018abc	1.741 ± 0.182g	2.833 ± 0.102cd
S9	22.167 ± 0.013bc	0.267 ± 0.2177bc	1.705 ± 0.048a	5.460 ± 0.154a	6.368 ± 0.041a	3.667 ± 0.079ab
CK	20.600 ± 0.026d	0.233 ± 0.247c	0.554 ±0.047d	3.690 ± 0.120cd	1.699 ± 0.105g	2.333 ± 0.124d

*n* = 3.

### 3.2. Effect of the inoculation with different PGPRs on the photosynthetic pigment content in the leaves of the P. Praeruptorum

The results show that compared with group CK, there was a considerable increase in the content of total chlorophyll, chlorophyll a and chlorophyll b in the leaves of *P. Praeruptorum* due to the inoculation of groups S1-S9 with PGPR, reaching a statistically significant extent (*P* < 0.05). Table 2 lists the results. According to the results of chlorophyll a/b, it was smaller in all treatment groups than in group CK, which was statistically significant (*P* < 0.05). The content of chlorophyll a in group S9 was the highest, reaching a level that was 1.06 times higher compared to group CK. Group S6 had the highest content of chlorophyll b and total chlorophyll, which was 1.87 times and 1.40 times higher than that in group CK, respectively. Compared to group CK, the inoculation of different PGPR significantly increased the content of photosynthetic pigment in *P. Praeruptorum* leaves, with the difference reaching a significant extent (*P* < 0.05). To be specific, the effect was most satisfactory in group S6, while the effect on the content of chlorophyll a was less significant.

**Table 2.** Effect of the inoculation with different PGPRs on the photosynthetic pigment content in the leaves of *P. Praeruptorum*.

Treatments	Chlorophyll a (mg/g)	Chlorophyll b (mg/g)	Chlorophyll a/b	Total Chlorophyll Content (mg/g)
S1	1.497 ± 0.023a	1.325 ± 0.083fg	1.136 ± 0.099ab	2.821 ± 0.029f
S2	1.434 ± 0.010a	1.807 ± 0.037bc	0.795 ± 0.038defg	3.241 ± 0.020c
S3	1.455 ± 0.009a	1.673 ± 0.119cd	0.878 ± 0.116cde	3.128 ± 0.062cd
S4	1.449 ± 0.003a	1.536 ± 0.061de	0.945 ± 0.059cd	2.985 ± 0.032de
S5	1.434 ±0.005a	2.007 ± 0.027b	0.715 ± 0.037fg	3.440 ± 0.012b
S6	1.438 ± 0.060a	2.250 ± 0.047a	0.641 ± 0.110g	3.689 ± 0.006a
S7	1.424 ± 0.009a	1.872 ± 0.029cd	0.757 ± 0.025efg	3.288 ± 0.019bc
S8	1.459 ± 0.006a	1.420 ± 0.045ef	1.029 ± 0.049bc	2.879 ± 0.021e
S9	1.509 ± 0.112a	1.790 ± 0.040c	0.846 ± 0.153def	3.299 ± 0.032bc
CK	1.417 ± 0.005a	1.203 ± 0.083g	1.189 ± 0.077a	2.627 ± 0.042f

*n* = 3.

### 3.3. Effect of the inoculation with different PGPRs on the activity of protective enzymes in the leaves of the P. Praeruptorum

As indicated by the results, different PGPR had different effects on the activities of protective enzymes in the leaves of *P. Praeruptorum* (Table 3). The results of CAT activity measurement show that the effect of inoculation with different PGPRs on CAT activity was more significant compared to group CK. More specifically, the activity of CAT in groups S3 and S9 was significantly stronger in comparison with group CK, reaching a level that was 3.36 times and 3.71 times higher than in group CK, respectively. According to the results of SOD activity, the activity of SOD in the leaves of *P. Praeruptorum* was significantly stronger *(P* < 0.05) than that in the CK group in all treatment groups except for the S6 group. Among them, the treatment group S5 exhibited the strongest SOD activity in the leaves of *P. Praeruptorum*, which was 2.32 times that of group CK. The result of POD activity show that the POD activity was significantly stronger in some treatment groups than in group CK (*P* < 0.05). Specifically, group S1 exhibited the strongest POD activity in the leaves of *P. Praeruptorum*, which was 5.94 times that of group CK. Overall, the inoculation with PGPR significantly enhanced the activity of protective enzymes in the leaves of *P. Praeruptorum*, with the effect reaching the optimal level in group S5.

**Table 3.** Effect of the inoculation with different PGPRs on the activity of protective enzymes in the leaves of *P. Praeruptorum*.

Treatments	CAT Activity/(U/g·min)	SOD Activity(U/g·min)	POD Activity/(U/g·min)
S1	397.112 ± 0.606b	0.201 ± 0.037d	297092.200 ± 0.135a
S2	829.491 ± 0.667ab	0.317 ± 0.047ab	52529.300 ± 0.028c
S3	1211.648 ± 0.318a	0.269 ± 0.009c	58143.300 ± 0.171c
S4	723.791 ± 0.415ab	0.302 ± 0.024b	175359.200 ± 0.391b
S5	836.697 ± 0.150ab	0.338 ± 0.037a	227008.100 ± 0.413ab
S6	437.990±0.166b	0.151±0.058g	50258.900±0.190c
S7	384.057 ± 0.027b	0.242 ± 0.018d	54948.900 ± 0.075c
S8	917.806 ± 0.449ab	0.174 ± 0.114f	219073.300 ± 0.471ab
S9	1339.791 ± 0.284a	0.309 ± 0.024b	191410.200 ± 0.037b
CK	360.734 ± 0.056b	0.146 ± 0.008g	50036.000 ± 0.206c

*n* = 3.

### 3.4. Effect of the inoculation with different PGPRs on the content of MDA, soluble protein and sugar in the leaves of P. Praeruptorum

As shown in Table 4, the content of MDA in the leaves of *P. Praeruptorum* was lower for all treatment groups than for the CK group without inoculation, with the difference reaching a statistically significant extent (*P* < 0.05). It is indicated that PGPR could effectively reduce the content of MDA in the leaves of *P. Praeruptorum* and alleviate the damage caused by lipid membrane peroxidation to plant cells. To be specific, group S6 had the lowest content of MDA in the leaves of *P. Praeruptorum*, which was only 1/3 that in group CK. According to the results of soluble sugar content determination, except for group S8, the content of soluble sugar in the leaves of *P. Praeruptorum* was higher to varying degrees in all other treatment groups than in group CK, and the difference reached a significant extent (*P* < 0.05). Group S2 had the highest content of soluble protein in the leaves of *P. Praeruptorum*, which was 3.82 times in contrast to the CK group. Overall, the inoculation with PGPR significantly reduced the content of MDA and effectively increased the content of osmoregulatory substances in the leaves of *P. Praeruptorum*. Consequently, cell membrane damage was alleviated and its resistance to stress was enhanced. Also, the effect was better in group S2.

**Table 4.** Effect of the inoculation with different PGPRs on the content of MDA, soluble sugar and protein in the leaves of *P. Praeruptorum*.

Treatments	MDA Content/(umol/g)	Soluble Sugar Content/(umol/g)	Soluble Protein Content/(mg/g)
S1	0.077 ± 0.020b	0.740 ± 0.513ab	38.763 ± 0.059c
S2	0.039 ± 0.141cd	0.784 ± 0.100a	98.909 ± 0.066a
S3	0.047 ± 0.075cd	0.569 ± 0.073abc	78.023 ± 0.075b
S4	0.059 ± 0.088bc	0.663 ± 0.198abc	34.964 ± 0.201c
S5	0.048 ± 0.079cd	0.778 ± 0.186a	36.199 ± 0.122c
S6	0.035 ± 0.092d	0.482 ± 0.016bc	93.375 ± 0.050a
S7	0.054 ± 0.015bcd	0.669 ± 0.070abc	65.682 ± 0.290b
S8	0.038 ± 0.107d	0.417 ± 0.086c	77.883 ± 0.051b
S9	0.042 ± 0.078cd	0.661 ± 0.101abc	66.967 ± 0.057b
CK	0.116 ± 0.295a	0.388 ± 0.190c	25.902 ± 0.080c

*n* = 3.

### 3.5. Correlation analysis

The physiological indicators used in the experiment were correlated with the photosynthetic pigment content in the leaves of *P. Praeruptorum*. According to the results shown in Table 5, there was a significant positive correlation between chlorophyll a content and POD activity (r = 0.711, *P* < 0.05). The content of chlorophyll b was negatively correlated with MDA content to a significant extent (r = −0.720, *P* < 0.05). The content of soluble sugar was positively associated with SOD activity (r = 0.795, *P* < 0.01). There was a significant positive correlation between soluble sugar content and SOD activity (r = 0.795, *P* < 0.01).

**Table 5.** Correlation analysis.

Physiological Indicators	Chlorophyll a Content	Chlorophyll b Content	MDA Content	Soluble Sugar Content	Soluble Protein Content	SOD Activity	CAT Activity	POD Activity
Chlorophyll a Content	1							
Chlorophyll b Content	−0.176	1						
MDA Content	−0.263	−0.720*	1					
Soluble Sugar Content	−0.084	0.078	−0.257	1				
Soluble Protein Content	0.088	0.588	−0.746*	−0.046	1			
SOD Activity	−0.191	0.073	−0.389	0.795**	−0.004	1		
CAT Activity	0.261	−0.032	−0.533	0.142	0.309	0.584	1	
POD Activity	0.711*	−0.263	−0.041	0.317	−0.441	0.165	0.148	1

“*” indicates a significant correlation between the two (*P* < 0.05). “**” indicates a very close correlation between the two (*P* < 0.01), *n* = 3.

### 3.6. Principal component analysis

The principal component of 14 indexes was carried out with the SPSS 25.0 software to fully demonstrate the effects of different PGPRs on the growth and physiological characteristics of *P. Praeruptorum*. As shown in Table 6, the cumulative contribution rate of the first five components extracted reaches 89.36%, which satisfies the analytical requirements. According to eigenvectors, the first principal component was affected by the indicators such as leaf area, leaf length, CAT activity and MDA content, with a contribution rate of 29.19%; the second principal component was affected by the indicators such as POD activity, stem diameter and chlorophyll b content, with a contribution rate of 21.50%; the third principal component was affected by leaf length, plant height and chlorophyll a content, with a contribution rate of 17.85%; the fourth principal component was affected by leaf width, SOD and CAT activities, with a contribution rate of 12.51%; the fifth principal component was affected by leaf area, POD and CAT activities, with a contribution rate of 8.32%. The value of each trait index for the five principal components was standardized to obtain the linear regression equation of the five principal components as follows:

$$F_{1}=0.404X_{1}-0.390X_{2}+0.355X_{3}+0.316X_{4}+0.290X_{5}+0.199X_{6}+0.105X_{7}+0.190X_{8}+0.188X_{9}+0.164X_{10}+0.199X_{11}+0.268X_{12}+0.241X_{13}+0.237X_{14}$$

$$F_{2}=0.052X_{1}+0.261X_{2}-0.023X_{3}+0.043X_{4}+0.318X_{5}+0.426X_{6}-0.409X_{7}-0.400X_{8}-0.379X_{9}+0.331X_{10}+0.104X_{11}-0.094X_{12}+0.204X_{13}-0.044X_{14}$$

$$F_{{\;}^{3}}=0.252X_{{\;}^{1}}+0.226X_{2}+0.319X_{3}-0.242X_{4}-0.066X_{5}+0.003X_{6}+0.126X_{7}-0.011X_{8}-0.282X_{9}-0.346X_{10}-0.526X_{11}+0.162X_{12}+0.312X_{13}+0.305X_{14}$$

$$F_{4}=0.072X_{1}-0.041X_{2}+0.238X_{3}-0.356X_{4}+0.103X_{5}-0.029X_{6}+0.127X_{7}-0.224X_{8}+0.159X_{9}+0.150X_{10}-0.037X_{11}-0.584X_{12}+0.437X_{13}+0.390X_{14}$$

$$F_{{\;}^{5}}=-0.358X_{{\;}^{1}}-0.121X_{2}-0.171X_{3}-0.348X_{4}-0.175X_{5}+0.452X_{6}+0.405X_{7}+0.109X_{8}-0.053X_{9}+0.379X_{10}-0.021X_{11}+0.051X_{12}+0.056X_{13}+0.379X_{14}$$

**Table 6.** Principal component matrix.

Indicators	Main Ingredient 1	Main Ingredient 2	Main Ingredient 3	Main Ingredient 4	Main Ingredient 5
Leaf area	0.816	0.091	0.398	0.095	−0.386
MDA	−0.788	0.454	0.357	−0.055	−0.130
Leaf length	0.717	−0.040	0.504	0.315	−0.185
CAT	0.639	0.074	−0.383	−0.471	−0.375
Petiole length	0.586	0.552	−0.105	0.137	−0.188
POD	0.403	0.740	0.003	−0.039	0.488
Stem diameter	0.213	−0.710	0.199	0.160	0.437
Chlorophyll b	0.385	−0.693	−0.176	0.296	0.118
Soluble protein	0.380	−0.658	−0.446	0.210	−0.058
Chlorophyll a	0.332	0.574	−0.547	0.198	0.409
Plant Height	0.403	0.181	−0.831	−0.048	−0.023
SOD	0.542	−0.163	0.256	−0.773	0.055
Leaf width	0.488	0.354	0.493	0.579	0.061
Soluble sugar	0.480	−0.076	0.482	−0.517	0.409
Eigenvalues	4.086	3.010	2.498	1.751	1.165
Contribution rate (%)	29.19%	21.50%	17.85%	12.51%	8.32%
Cumulative contribution rate (%)	29.19%	50.69%	68.53%	81.04%	89.36%

*n* = 3.

In the formula, *X*_1_~*X*_14_ represent leaf length, leaf width, leaf area, stem diameter, petiole length, and plant height; CAT, SOD, POD activities; chlorophyll a, chlorophyll b, MDA, soluble sugar, soluble protein content. According to the established linear regression equation, five principal component scores (*F*_*1*_, *F*_*2*_, *F*_*3*_, *F*_*4*_, *F*_*5*_) and the evaluation model used for (*F* = 0.291*F*_*1*_ + 0.215*F*_*2*_ + 0.178*F*_*3*_ + 0.125*F*_*4*_ + 0.083*F*_*5*_) evaluating the comprehensive score of PGPR vaccination effect were obtained. As shown in Table 7, the growth-promoting effect of different PGPR on *P. Praeruptorum* was evaluated in the following order: S9, S1, S5, S6, S4, S8, S3, S7, S2. Among them, group S9 had the highest comprehensive score in growth-promoting effect, which indicates the best outcome of treatment.

**Table 7.** Principal component scores.

No.	*F1*	Scores	*F2*	Scores	*F3*	Scores	*F4*	Scores	*F5*	Scores	*F*	Scores
S1	0.116	5	3.066	1	0.926	3	0.730	2	1.453	1	1.070	2
S2	−0.061	6	−2.951	10	−0.128	8	−1.521	10	0.985	2	−0.784	9
S3	0.512	4	0.138	6	−1.872	9	−1.043	8	−1.524	10	−0.412	7
S4	−0.246	8	0.028	7	1.287	1	−0.634	7	0.888	3	0.158	5
S5	1.547	2	0.215	5	0.664	5	−1.240	9	0.215	5	0.479	3
S6	0.983	3	−2.074	9	0.436	7	3.062	1	−0.382	7	0.270	4
S7	−1.136	9	−1.302	8	0.884	4	0.160	4	0.060	6	−0.429	8
S8	−0.102	7	0.749	4	−3.715	10	0.668	3	0.874	4	−0.375	6
S9	3.096	1	1.248	2	0.959	2	−0.193	6	−1.412	9	1.202	1
CK	−4.710	10	0.884	3	0.559	6	0.010	5	−1.158	8	−1.180	10

*n*=3.

## 4 Discussion

As a new type of biological fertilizer, PGPR could promote the absorption and utilization of nutrients by plants, which is effective not only in improving the soil microenvironment,^29^ but also in enhancing the stress resistance of plants through its own action or the production of various metabolites.^30,31^ Thus, the growth of plants is promoted, crop quality is improved and economic benefits are boosted. As revealed by this study, 9 treatment groups showed a significant improvement in the growth indexes of *P. Praeruptorum* compared with group CK. Overall, group S9 performed best. This is because PGPR is capable of producing growth hormone (IAA) and gibberellin (GA), both of which promote plant growth indirectly.^14^

Leaf is the primary organ where photosynthesis occurs for plants, and the size of leaf area is reflective of the light-receiving area on plants.^32^ When the light-receiving area of the plants is expanded, its photosynthesis is enhanced, thus promoting plant development.^32^ In this study, the leaf area was significantly larger in all treatment groups than in group CK (*P* < 0.05). As an important way for plants to synthesize organic compounds, photosynthesis plays a key role in the growth of plants. Photosynthetic pigments are essential for photosynthesis, whose content is closely related to the photosynthetic capacity of plants.^33^ In the present study, it is found out that the inoculation with PGPR could effectively increase the content of photosynthetic pigment in the leaves of *P. Praeruptorum*. Group S6 had the highest level of photosynthetic pigment content, which indicates that the inoculation with PGPR could accelerate the process of photosynthesis, thus promoting the accumulation of organic substances and the development of *P. Praeruptorum*.

The content of MDA reflects the severity of damage caused to plant cell membranes and their performance in stress resistance. The lower the level of MDA content, the more stable the cell membrane system and the stronger the stress resistance of plants.^34^ This study reveals that PGPR could be effective in reducing the content of MDA in the leaves of *P. Praeruptorum*. To be specific, the content of MDA was the lowest in group S6. The antioxidant enzyme system is capable to remove reactive oxygen species from plants, maintain the normal physiological functions and improve the stress resistance of plants.^17,35^ The greater its activity, the stronger the stress resistance of the plant.^32^ It is confirmed in this study that the inoculation with PGPR significantly enhanced CAT, POD and SOD activities. As osmotic regulatory substances, soluble sugar and protein affect the stress resistance of plants.^36^ Through experiment, it was found out that the content of soluble sugar and protein in each treatment group was higher compared to group CK. In general, group S2 was the best. In summary, the inoculation of PGPR reduced the content of MDA in the leaves of *P. Praeruptorum*, thus alleviating the damage caused to cell membrane. The osmotic pressure balance of cell membranes can be maintained by increasing the content of osmotic regulation substances and enhancing the activity of antioxidant enzymes, which improves the stress resistance of *P. Praeruptorum*.

## 5 Conclusion

In summary, the inoculation with different PGPRs promoted the growth and photosynthesis of *P. Praeruptorum* to varying degrees, thus improving its environmental resistance. In this study, the comprehensive inoculation effect was screened by conducting principal component analysis, with the results showing that compared with other treatment groups, the inoculation effect was more significant in groups S9 (*B. wiedmannii*), S5 (*B. aryabhattai*), S1 (*B. thuringiensis*) and S6 (*B. cereus*) groups. Among them, the treatment group S9 (*B. wiedmannii*) performed best. Therefore, it is presumed that PGPR is applicable to the large-scale cultivation of *P. Praeruptorum* for the improved quality and economic benefits of *P. Praeruptorum*, which lays a solid foundation for cultivating high-quality *P. Praeruptorum* and provides theoretical reference for the healthy development of the *P. Praeruptorum* industry.
